# Event and Comment

**Published:** 1919-12

**Authors:** 


### DENTAL REGISTER

THE MAGAZINE OF DENTISTRY

Vol. LXXIII DECEMBER, 1919 No. 12

### EVENT AND COMMENT

**Christmas in America** Ranging above the fireplace, or along the wall, hang the stockings, all in a row. Baby Joe's is there—a tiny knitted little thing of silk, perhaps, and doesn't it look funny when compared with dad's, suspended boldly at the other end of the row! Every stocking, small or large, is loaded to capacity. A rattle for Baby Joe, with other things to catch the baby eye and ear; a brand new pair of skates for Johnnie Boy, with oranges, nuts, candies, etc.

And so, all along the line up to where daddy's mighty foot-gear bulges out with an odd assortment of almost everything, Christmas gifts await the happy family.

**Christmas in Armenia** Far away, across the ocean, in the Bible lands made sacred by the footsteps of the Savior, whose birthday we commemorate, other little children awake on this same Christmas morn to find—gifts? No, not even stockings to put them in if there were gifts—not even stockings to wear! The fireplace is cold and dreary. Down its bleak and cheerless chimney no jolly Santa Claus has ventured.

If Santa Claus should come that way, what do you suppose would be the dearest treasure in his pack?

Bread, only bread—that, to thousands of little, starving tots, would be the greatest boon of all on Christmas morn!

Send a gift through *The Near East Relief,* 1 Madison Avenue, New York City.

**Is Real Preventive Dentistry a Practicable Possibility?** So much has been said and written about preventive dentistry, and so little effort has been put forth in the way of accomplishing it, that we often wonder if there is any possibility of its ever materializing. Both medical and dental writers are constantly referring to preventive medicine and preventive dentistry in such casual and careless terms as to leave the impression that they have no definite conception of any well-developed scheme for accomplishing this most desirable condition of health. The following quotation from an article by Dr. Edward C. Rosenow, in the November issue of *The Journal of the National Dental Association*, makes a very pertinent suggestion which, it seems to us, comes nearer defining what preventive dentistry should be than anything we have seen or heard before, although it is not a new or ideal suggestion. But it is at least encouraging to have the idea proclaimed by so eminent a medical research authority as Dr. Rosenow. He says: "The opportunity for the dental profession to co-operate with the medical profession along therapeutic lines needs no emphasizing. The prevention of oral sepsis in the future, with a view to lessening the incidence of systemic disease, should henceforth take precedence, in dental practice, over the preservation of the teeth almost wholly for mechanical or cosmetic purposes, as has been so largely the case in the past. Every effort should be made for the prevention of dental infections and for the correction of those already present. Preventive measures should begin in childhood with the idea in view of obtaining perfect development of the teeth and oral cavity, and thus prevent various defects which would later lead to sepsis. This calls for co-operation of the dentist, the pediatrist, and the throat specialist."

While this statement does not tell us specifically how this may be accomplished, the fact that one who has spent so much time and produced so much accurate knowledge of the way in which dental diseases have become involved with systemic diseases of the most fatal character, his suggestion that dentistry has a more important function than mere technical rescue and restoration procedures, is worthy of careful consideration. If we stop to think of the dental situation of to-day, we can easily determine, in spite of the fact that we have made tremendous advances in technical art, that we are making little progress in preventing the existence of the most universally prevalent disease afflicting mankind. And what is still a more grave discovery is that we as a profession have unknowingly contributed to systemic diseases through our technical art, which we have pursued so persistently, that we have failed to discover its important relations and obligations to other tissues and organs of the body. Dr. Rosenow strikes the right key to the situation when he says that we must give more attention to the development of the dental organs and tissues that they may have greater power to resist the degenerating influences to which the human teeth are subjected. It is true that we have recognized this fact for many years, but we have not made it our chief anxiety, nor has it been our ambition to organize a better defense against the encroachment of dental caries, even though we have had for some years a good knowledge of how it is caused.

Miller gave us a rational theory explaining caries of the teeth, and he did all that he could to show us the way out, and possibly he would have led us out had he lived. Now, that we are brought to realize the seriousness of our profession, is it not high time that we do some good thinking, and start on another course that will have for its goal the perfectly developed human denture as a probable help in obtaining something better than we now have in the way of an antidote for dental caries? It seems easily possible to make a beginning for practicable prevention if we could start with the first teeth that the child develops, by instituting in the very beginning a strict regimen of oral prophylaxis. We have demonstrated the benefits of this treatment in all ages, but we have as yet never tested it out on children, starting with the first tooth erupted and continuing it through childhood until all the permanent teeth are fully erupted. We believe, if this work was carefully followed out until the child has reached the age of sixteen without ever having experienced a toothache, that we should have a better developed permanent set of teeth, and the child would have a firmly-established habit of oral hygiene that would prevent the development of caries during the next twenty-five years at least, with the ordinary care that such patients would have learned to give their teeth. If we could carry our patients to the age of say forty years, with a minimum of injury to the integrity of their teeth, there would be every reason to expect that such patients would never become entirely edentulous, or be the victims of focal infections. The dental profession has been notoriously negligent in serving our little patients at a time when the most careful treatment would count more for health of the teeth as well as the body than at any other period of their existence. And now, since the medical profession has condemned to annihilation all dental organs whose pulps have become infected, which will result in the loss of most of the teeth of all patients over sixty years of age, and many who have not passed their thirtieth birthdays, would it not be a wise program to put all the available professional force to work in an effort to conserve the children's teeth? By doing only such rescue treatment for the older patients as shall make them reasonably comfortable and able to get through life the best they can, and by giving better care to the future men and women, we could hope to have a healthier and more capable race of people.

**Dental Educational Requirements in New York** Dental students and members of the profession, expecting to locate for practice in New York State, will be interested to know of the present and future requirements for practice in that state.

The present requirements are a certificate of graduation from a registered high school or an equivalent education (72 Regent's counts) approved by the Examinations Division of the New York State Education Department. The high school diploma is not sufficient unless evidence be submitted, showing the satisfactory completion in an approved school of a one-year course in each of the following sciences: physics, chemistry and biology; or, in lieu thereof, the passing of each of these sciences at 75% or above in Regent's examinations.

After January 1, 1921, a "Dental Student Qualifying Certificate" may only be obtained upon the presentation of satisfactory evidence of the completion of not less than one year of instruction in any approved college of liberal arts and science, after the completion of an approved four-year high school course, based upon eight years of elementary preparation. The year of college instruction must be of at least fifteen week hours, including English 3, physics 3, biology 3, chemistry 3, and 3 optional.

---

**Death of William W. Belcher** Dr. Belcher, the editor of *Oral Hygiene*, died at his home in Rochester, N. Y., Saturday morning, December 4th. Dr. Belcher has not been in good health for some time, but had gained in strength so much this fall that his friends were encouraged to believe that he would soon be fully restored to good health. Dr. Belcher had a host of friends, who will be shocked and grieved to learn of his death. His genial and friendly attitude to every one made him a general favorite, and few men in the profession had such cordial relations with their associates.

Dr. Belcher became interested in the oral hygiene movement and was very active in organizing the first dental clinic to care for the children's teeth in the city of Rochester, February 22, 1905. He maintained this interest to the day of his death; in fact, it was almost an obsession with him. When Dr. George E. Hunt, the editor of *Oral Hygiene,* died August, 1914, Dr. Belcher succeeded him and has edited *Oral Hygiene* with marked ability and characteristic devotion to the cause of oral hygiene to the day of his death. In his salutatory editorial, he said his "job was to make *Oral Hygiene* the most readable magazine in the dental field," and he succeeded better than he feared he might. It certainly is read by more dentists than any dental magazine ever published, and Dr. Belcher has given character to the reading matter that has kept the oral hygiene idea constantly before the dental profession. He was just starting a campaign of public education in dental matters that promised to be epoch-making in results. It seems sad that he could not have lived to realize the results of this new work and share in the glory of its achievement.

---

**Things to do in 1920** Subscribe for and read at least three dental journals.

Join the Red Cross Society and help the world's unfortunates to get some joy out of life.

Resolve to attend your local or district and also your State Dental Society meetings.

Send a gift to The Near East Relief Society, 1 Madison Ave., New York City, for the Armenians.

Buy and read at least one recent text-book on some dental subject.

Send a contribution to the Dental Relief Committee for Aged and Destitute Dentists.

Join a study club if practicable, or organize one in your own neighborhood if you can induce one or more dentists to join you.

Strive to get all men to live more unselfishly and to speedily restore peace on the earth.

